# 2-Methyl-1-(phenyl­sulfon­yl)naphtho[2,1-*b*]furan

**DOI:** 10.1107/S1600536808007046

**Published:** 2008-03-20

**Authors:** Hong Dae Choi, Pil Ja Seo, Byeng Wha Son, Uk Lee

**Affiliations:** a Department of Chemistry, Dongeui University, San 24 Kaya-dong, Busanjin-gu, Busan 614-714, Republic of Korea; b Department of Chemistry, Pukyong National University, 599-1 Daeyeon 3-dong, Nam-gu, Busan 608-737, Republic of Korea

## Abstract

The title compound, C_19_H_14_O_3_S, was prepared by the oxidation of 2-methyl-1-(phenyl­sulfan­yl)naphtho[2,1-*b*]furan with 3-chloro­peroxy­benzoic acid. The phenyl ring makes a dihedral angle of 87.13 (4)° with the plane of the naphthofuran fragment. The crystal structure is stabilized by π–π inter­actions between the furan and benzene rings of neighbouring mol­ecules [centroid–centroid distance = 3.850 (2) Å] and weak C—H⋯π inter­actions. In addition, there are also intra­molecular C—H⋯O inter­actions.

## Related literature

For the crystal structures of similar 2-methyl­naphtho[2,1-*b*]furan compounds, see: Choi *et al.* (2006 ▶, 2007 ▶). 
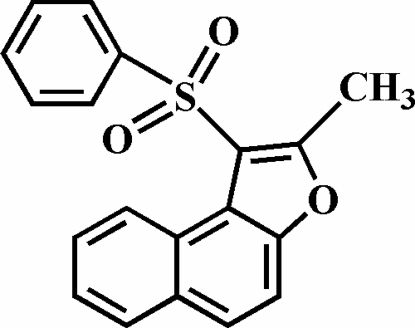


## Experimental

### 

#### Crystal data

C_19_H_14_O_3_S
*M* *_r_* = 322.36
Monoclinic, 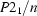

*a* = 10.7175 (4) Å
*b* = 7.7972 (3) Å
*c* = 18.0488 (7) Å
β = 97.797 (1)°
*V* = 1494.33 (10) Å^3^
*Z* = 4
Mo *K*α radiation
μ = 0.23 mm^−1^
*T* = 173 (2) K
0.40 × 0.40 × 0.20 mm

#### Data collection

Bruker SMART CCD diffractometer
Absorption correction: none
8784 measured reflections
3222 independent reflections
2785 reflections with *I* > 2σ(*I*)
*R*_int_ = 0.023

#### Refinement

*R*[*F* ^2^ > 2σ(*F* ^2^)] = 0.036
*wR*(*F* ^2^) = 0.098
*S* = 1.05
3222 reflections
210 parameters
H-atom parameters constrained
Δρ_max_ = 0.33 e Å^−3^
Δρ_min_ = −0.39 e Å^−3^

### 

Data collection: *SMART* (Bruker, 2001 ▶); cell refinement: *SAINT* (Bruker, 2001 ▶); data reduction: *SAINT*; program(s) used to solve structure: *SHELXS97* (Sheldrick, 2008 ▶); program(s) used to refine structure: *SHELXL97* (Sheldrick, 2008 ▶); molecular graphics: *ORTEP-3* (Farrugia, 1997 ▶) and *DIAMOND* (Brandenburg, 1998 ▶); software used to prepare material for publication: *SHELXL97*.

## Supplementary Material

Crystal structure: contains datablocks global, I. DOI: 10.1107/S1600536808007046/fb2090sup1.cif

Structure factors: contains datablocks I. DOI: 10.1107/S1600536808007046/fb2090Isup2.hkl

Additional supplementary materials:  crystallographic information; 3D view; checkCIF report

## Figures and Tables

**Table 1:** Hydrogen-bond geometry (Å, °)

*D*—H⋯*A*	*D*—H	H⋯*A*	*D*⋯*A*	*D*—H⋯*A*
C4—H4⋯O2	0.95	2.35	3.190 (2)	147
C18—H18⋯O2	0.95	2.46	2.869 (2)	106
C19—H19*B*⋯O3	0.98	2.55	2.926 (2)	103
C19—H19*C*⋯*Cg*3^i^	0.98	3.03	3.735 (3)	130
C16—H16⋯*Cg*3^ii^	0.95	2.88	3.761 (3)	155

Symmetry codes: (i) 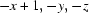; (ii) 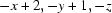. *Cg*3 is the centroid of the benzene ring of the naphthofuran unit.

**Fig. 1.**
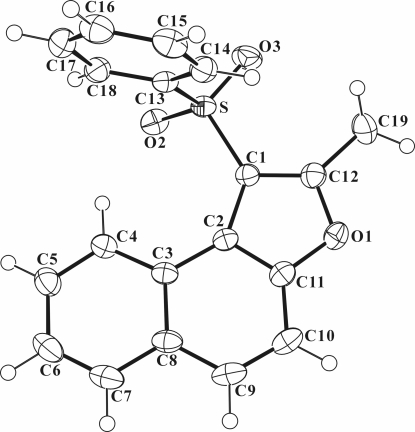
The molecular structure of the title compound, showing displacement ellipsoides drawn at the 50% probability level.

**Fig. 2.**
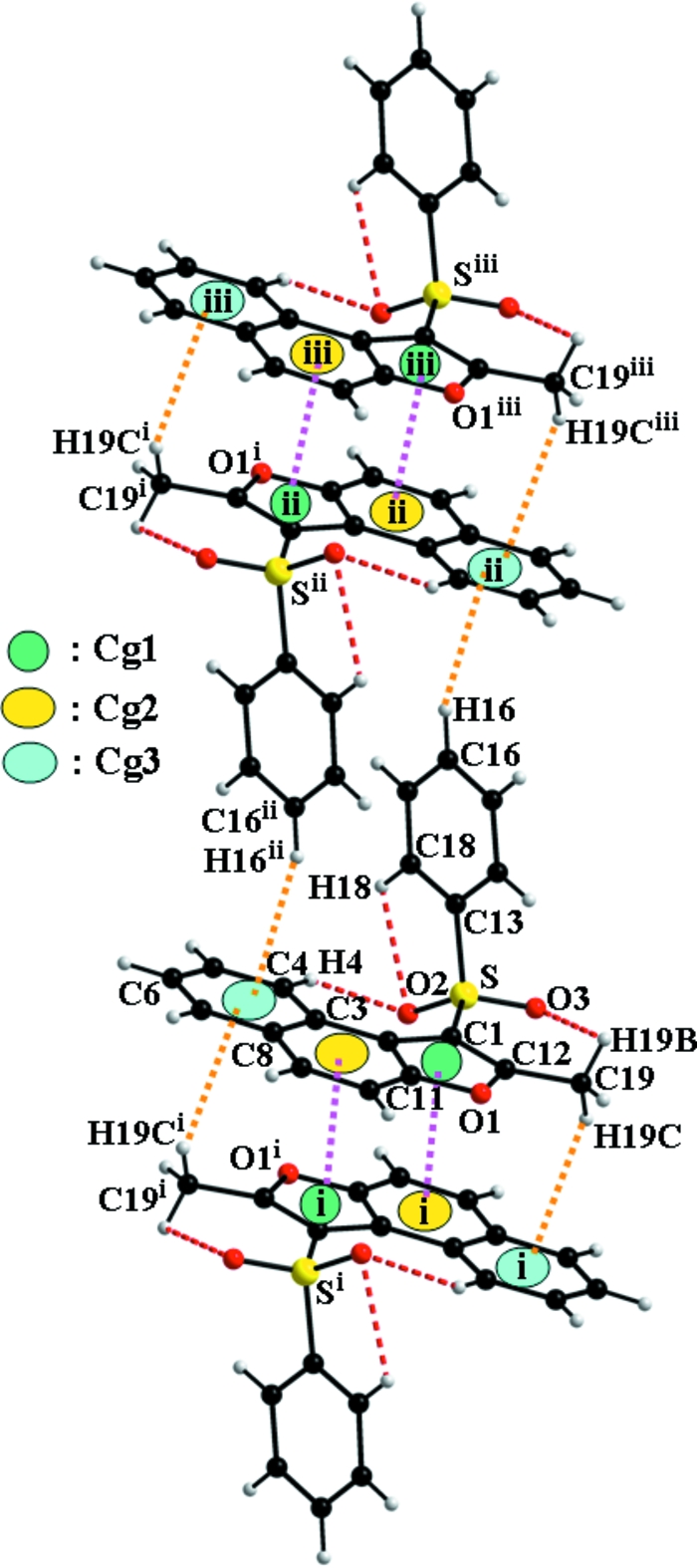
π—π, C—H···π and intramolecular C—H···O interactions (dotted lines) in the title compound. Cg denotes the ring centroids. [Symmetry code: (i) 1 - x, -y, -z; (ii) 2 - x, 1 - y, -z; (iii) x + 1, y + 1, z.]

## supplementary crystallographic information

### Comment

As a part of our ongoing studies on the synthesis and structure of 2-methylnaphtho[2,1-*b*]furan derivatives, the crystal structures of 2-methyl-1-(methylsulfinyl)naphtho[2,1-*b*]furan (Choi *et al.*, 2006) and 2-methyl-1-(phenylsulfinyl)naphtho[2,1-*b*]furan (Choi *et al.*, 2007) have already been described. Herein we report the molecular and the crystal structure of the title compound, 2-methyl-1-(phenylsulfonyl)naphtho[2,1-*b*]furan (Fig. 1).

The naphthofuran unit is essentially planar, with a mean deviation equal to 0.040 Å from the least-squares plane defined by thirteen constituent atoms. The crystal packing (Fig. 2) is stabilized by aromatic π—π stacking interactions between the furan and the benzene rings from the adjacent naphthofuran fragments. The *Cg*1···*Cg*2^i^ distance is 3.850 (2) Å (*Cg*1 and *Cg*2 are the centroids of the O1/C12/C1/C2/C11 furan and the C2/C3/C8/C9/C10/C11 benzene rings, respectively, the symmetry code as in Fig. 2). The crystal packing (Fig. 2) is further stabilized by the C—H···π interactions; in both cases the benzene ring of the naphthofuran unit (*Cg*3) is involved. There are also intramolecular C—H···O interactions present in the structure.

### Experimental

3-Chloroperoxybenzoic acid (77%, 471 mg, 2.10 mmol) was added in small portions to a stirred solution of 2-methyl-1-(phenylsulfanyl)naphtho[2,1-*b*]furan (290 mg, 1.0 mmol) in dichloromethane (20 ml) at 273 K. After having been stirred for 4 h at room temperature, the mixture was washed with saturated sodium hydrogencarbonate solution and the organic layer was separated, dried over magnesium sulfate, filtered and concentrated in vacuum. The residue was purified by column chromatography (hexane-ethyl acetate, 2:1 *v*/*v*) to afford the title compound as a colourless solid [yield 84%, m.p. 431–432 K; *R*_f_ = 0.63 (hexane-ethyl acetate, 2:1 *v*/*v*)]. Single crystals suitable for X-ray diffraction were prepared by slow evaporation of a solution of the title compound in acetone at room temperature. The average crystal size was approximately 1.0 × 1.0 × 0.5 mm. The crystals are colourless and soluble in polar solvents.

### Refinement

All the H atoms were discernible in the difference Fourier map. Nevertheless, during the refinement the H atoms were positioned into idealized positions and refined using a riding model with the distance constraints: C—H = 0.95 Å for aryl H atoms and 0.98 Å for methyl H atoms, respectively, and with *U*_iso_(H) = 1.2Ueq(C) and 1.5Ueq(C) for aryl and methyl H atoms, respectively.

### Crystal data

**Table:**

C_19_H_14_O_3_S	*F*_000_ = 672
*M**_r_* = 322.36	*D*_x_ = 1.433 Mg m^−^^3^
Monoclinic, *P*2_1_/*n*	Melting point = 431–432 K
Hall symbol: -P 2yn	Mo *K*α radiation λ = 0.71073 Å
*a* = 10.7175 (4) Å	Cell parameters from 5468 reflections
*b* = 7.7972 (3) Å	θ = 2.3–28.2º
*c* = 18.0488 (7) Å	µ = 0.23 mm^−^^1^
β = 97.797 (1)º	*T* = 173 (2) K
*V* = 1494.33 (10) Å^3^	Block, colourless
*Z* = 4	0.40 × 0.40 × 0.20 mm

### Data collection

**Table:**

Bruker SMART CCD diffractometer	3222 independent reflections
Radiation source: fine-focus sealed tube	2785 reflections with *I* > 2σ(*I*)
Monochromator: graphite	*R*_int_ = 0.023
Detector resolution: 10.0 pixels mm^-1^	θ_max_ = 27.0º
*T* = 173(2) K	θ_min_ = 2.9º
φ and ω scans	*h* = −13→11
Absorption correction: none	*k* = −9→9
8784 measured reflections	*l* = −23→23

### Refinement

**Table:**

Refinement on *F*^2^	Secondary atom site location: difference Fourier map
Least-squares matrix: full	Hydrogen site location: difference Fourier map
*R*[*F*^2^ > 2σ(*F*^2^)] = 0.036	H-atom parameters constrained
*wR*(*F*^2^) = 0.098	*w* = 1/[σ^2^(*F*_o_^2^) + (0.0464*P*)^2^ + 0.7406*P*] where *P* = (*F*_o_^2^ + 2*F*_c_^2^)/3
*S* = 1.05	(Δ/σ)_max_ < 0.001
3222 reflections	Δρ_max_ = 0.33 e Å^−^^3^
210 parameters	Δρ_min_ = −0.39 e Å^−^^3^
54 constraints	Extinction correction: SHELXL97 (Sheldrick, 2008), Fc^*^=kFc[1+0.001xFc^2^λ^3^/sin(2θ)]^-1/4^
Primary atom site location: structure-invariant direct methods	Extinction coefficient: 0.015 (2)

### Special details

**Table:**

Geometry. All e.s.d.'s (except the e.s.d. in the dihedral angle between two l.s. planes) are estimated using the full covariance matrix. The cell e.s.d.'s are taken into account individually in the estimation of e.s.d.'s in distances, angles and torsion angles; correlations between e.s.d.'s in cell parameters are only used when they are defined by crystal symmetry. An approximate (isotropic) treatment of cell e.s.d.'s is used for estimating e.s.d.'s involving l.s. planes.
Refinement. Refinement of *F*^2^ against ALL reflections. The weighted *R*-factor *wR* and goodness of fit *S* are based on *F*^2^, conventional *R*-factors *R* are based on *F*, with *F* set to zero for negative *F*^2^. The threshold expression of *F*^2^ > σ(*F*^2^) is used only for calculating *R*-factors(gt) *etc*. and is not relevant to the choice of reflections for refinement. *R*-factors based on *F*^2^ are statistically about twice as large as those based on *F*, and *R*- factors based on ALL data will be even larger.

### Fractional atomic coordinates and isotropic or equivalent isotropic displacement parameters (Å^2^)

**Table:**

	*x*	*y*	*z*	*U*_iso_*/*U*_eq_	
S	0.78568 (4)	0.07420 (5)	0.11326 (2)	0.02579 (13)	
O1	0.44773 (10)	0.27156 (15)	0.07186 (6)	0.0317 (3)	
O2	0.81955 (11)	−0.07018 (14)	0.07052 (7)	0.0327 (3)	
O3	0.78345 (12)	0.05031 (17)	0.19220 (6)	0.0376 (3)	
C1	0.63938 (14)	0.1558 (2)	0.07427 (8)	0.0251 (3)	
C2	0.59348 (14)	0.19921 (19)	−0.00332 (8)	0.0239 (3)	
C3	0.63656 (14)	0.18767 (19)	−0.07498 (8)	0.0246 (3)	
C4	0.74995 (16)	0.1091 (2)	−0.08857 (9)	0.0294 (3)	
H4	0.8031	0.0575	−0.0483	0.035*	
C5	0.78480 (18)	0.1059 (2)	−0.15919 (9)	0.0360 (4)	
H5	0.8612	0.0516	−0.1671	0.043*	
C6	0.70826 (19)	0.1822 (3)	−0.21959 (9)	0.0407 (4)	
H6	0.7339	0.1820	−0.2680	0.049*	
C7	0.59747 (18)	0.2564 (2)	−0.20867 (9)	0.0383 (4)	
H7	0.5460	0.3066	−0.2500	0.046*	
C8	0.55669 (15)	0.2608 (2)	−0.13718 (9)	0.0297 (4)	
C9	0.43790 (16)	0.3342 (2)	−0.12790 (10)	0.0355 (4)	
H9	0.3867	0.3809	−0.1702	0.043*	
C10	0.39621 (15)	0.3389 (2)	−0.06035 (10)	0.0335 (4)	
H10	0.3164	0.3859	−0.0543	0.040*	
C11	0.47661 (14)	0.2710 (2)	0.00014 (9)	0.0273 (3)	
C12	0.54835 (15)	0.2028 (2)	0.11655 (9)	0.0294 (3)	
C13	0.89432 (14)	0.24061 (19)	0.10214 (8)	0.0247 (3)	
C14	0.87639 (16)	0.4045 (2)	0.12958 (9)	0.0299 (4)	
H14	0.8035	0.4308	0.1520	0.036*	
C15	0.96700 (17)	0.5283 (2)	0.12344 (9)	0.0351 (4)	
H15	0.9550	0.6417	0.1403	0.042*	
C16	1.07529 (17)	0.4877 (2)	0.09275 (9)	0.0367 (4)	
H16	1.1377	0.5730	0.0897	0.044*	
C17	1.09280 (16)	0.3244 (2)	0.06666 (10)	0.0360 (4)	
H17	1.1675	0.2969	0.0463	0.043*	
C18	1.00096 (15)	0.2003 (2)	0.07027 (9)	0.0301 (4)	
H18	1.0112	0.0886	0.0510	0.036*	
C19	0.53353 (18)	0.1975 (3)	0.19698 (10)	0.0414 (4)	
H19A	0.4597	0.2651	0.2054	0.062*	
H19B	0.6089	0.2455	0.2266	0.062*	
H19C	0.5222	0.0784	0.2121	0.062*	

### Atomic displacement parameters (Å^2^)

**Table:**

	*U*^11^	*U*^22^	*U*^33^	*U*^12^	*U*^13^	*U*^23^
S	0.0269 (2)	0.0252 (2)	0.0241 (2)	0.00073 (15)	−0.00082 (14)	0.00240 (14)
O1	0.0246 (6)	0.0341 (6)	0.0373 (6)	−0.0007 (5)	0.0076 (5)	0.0014 (5)
O2	0.0342 (6)	0.0231 (6)	0.0394 (6)	0.0013 (5)	0.0005 (5)	−0.0007 (5)
O3	0.0411 (7)	0.0446 (7)	0.0259 (6)	−0.0008 (6)	−0.0007 (5)	0.0089 (5)
C1	0.0243 (7)	0.0255 (8)	0.0250 (7)	−0.0014 (6)	0.0017 (6)	0.0009 (6)
C2	0.0226 (7)	0.0204 (7)	0.0276 (8)	−0.0032 (6)	−0.0001 (6)	0.0003 (6)
C3	0.0267 (7)	0.0214 (7)	0.0245 (7)	−0.0049 (6)	−0.0012 (6)	−0.0004 (6)
C4	0.0317 (8)	0.0298 (8)	0.0261 (8)	−0.0004 (6)	0.0017 (6)	−0.0011 (6)
C5	0.0386 (9)	0.0395 (10)	0.0307 (8)	−0.0018 (7)	0.0074 (7)	−0.0055 (7)
C6	0.0503 (11)	0.0482 (11)	0.0236 (8)	−0.0117 (9)	0.0056 (7)	−0.0026 (8)
C7	0.0438 (10)	0.0421 (10)	0.0262 (8)	−0.0096 (8)	−0.0061 (7)	0.0056 (7)
C8	0.0308 (8)	0.0282 (8)	0.0278 (8)	−0.0075 (7)	−0.0046 (6)	0.0032 (6)
C9	0.0304 (9)	0.0335 (9)	0.0386 (9)	−0.0035 (7)	−0.0093 (7)	0.0097 (7)
C10	0.0220 (8)	0.0305 (9)	0.0463 (10)	−0.0009 (6)	−0.0017 (7)	0.0061 (7)
C11	0.0240 (8)	0.0245 (8)	0.0331 (8)	−0.0040 (6)	0.0030 (6)	0.0009 (6)
C12	0.0271 (8)	0.0302 (8)	0.0311 (8)	−0.0032 (6)	0.0043 (6)	0.0011 (7)
C13	0.0249 (7)	0.0249 (8)	0.0228 (7)	0.0008 (6)	−0.0024 (6)	0.0007 (6)
C14	0.0316 (8)	0.0280 (8)	0.0293 (8)	0.0053 (6)	0.0015 (6)	−0.0006 (6)
C15	0.0471 (10)	0.0254 (8)	0.0311 (8)	−0.0008 (7)	−0.0009 (7)	−0.0016 (7)
C16	0.0410 (10)	0.0359 (10)	0.0318 (8)	−0.0107 (8)	0.0003 (7)	0.0023 (7)
C17	0.0304 (9)	0.0425 (10)	0.0355 (9)	−0.0028 (7)	0.0065 (7)	−0.0031 (8)
C18	0.0290 (8)	0.0297 (8)	0.0309 (8)	0.0021 (6)	0.0015 (6)	−0.0058 (7)
C19	0.0415 (10)	0.0521 (12)	0.0335 (9)	−0.0013 (9)	0.0154 (8)	0.0001 (8)

### Geometric parameters (Å, °)

**Table:**

S—O2	1.4387 (12)	C8—C9	1.426 (2)
S—O3	1.4403 (12)	C9—C10	1.355 (2)
S—C1	1.7492 (15)	C9—H9	0.9500
S—C13	1.7729 (16)	C10—C11	1.400 (2)
O1—C12	1.366 (2)	C10—H10	0.9500
O1—C11	1.3713 (19)	C12—C19	1.482 (2)
C1—C12	1.367 (2)	C13—C18	1.383 (2)
C1—C2	1.460 (2)	C13—C14	1.393 (2)
C2—C11	1.381 (2)	C14—C15	1.384 (2)
C2—C3	1.434 (2)	C14—H14	0.9500
C3—C4	1.412 (2)	C15—C16	1.389 (3)
C3—C8	1.434 (2)	C15—H15	0.9500
C4—C5	1.376 (2)	C16—C17	1.379 (3)
C4—H4	0.9500	C16—H16	0.9500
C5—C6	1.405 (3)	C17—C18	1.388 (2)
C5—H5	0.9500	C17—H17	0.9500
C6—C7	1.359 (3)	C18—H18	0.9500
C6—H6	0.9500	C19—H19A	0.9800
C7—C8	1.418 (2)	C19—H19B	0.9800
C7—H7	0.9500	C19—H19C	0.9800
			
Cg1···Cg2^i^	3.850 (2)		
			
O2—S—O3	117.93 (7)	C9—C10—C11	116.63 (15)
O2—S—C1	110.28 (7)	C9—C10—H10	121.7
O3—S—C1	108.10 (7)	C11—C10—H10	121.7
O2—S—C13	107.14 (7)	O1—C11—C2	111.47 (13)
O3—S—C13	107.81 (7)	O1—C11—C10	122.77 (14)
C1—S—C13	104.76 (7)	C2—C11—C10	125.75 (15)
C12—O1—C11	107.16 (12)	O1—C12—C1	109.97 (14)
C12—C1—C2	107.47 (14)	O1—C12—C19	114.10 (14)
C12—C1—S	122.78 (12)	C1—C12—C19	135.93 (16)
C2—C1—S	129.59 (11)	C18—C13—C14	121.17 (15)
C11—C2—C3	118.11 (14)	C18—C13—S	118.24 (12)
C11—C2—C1	103.91 (13)	C14—C13—S	120.44 (12)
C3—C2—C1	137.99 (14)	C15—C14—C13	118.61 (15)
C4—C3—C2	125.03 (14)	C15—C14—H14	120.7
C4—C3—C8	118.22 (14)	C13—C14—H14	120.7
C2—C3—C8	116.75 (14)	C14—C15—C16	120.44 (16)
C5—C4—C3	121.14 (15)	C14—C15—H15	119.8
C5—C4—H4	119.4	C16—C15—H15	119.8
C3—C4—H4	119.4	C17—C16—C15	120.42 (16)
C4—C5—C6	120.51 (17)	C17—C16—H16	119.8
C4—C5—H5	119.7	C15—C16—H16	119.8
C6—C5—H5	119.7	C16—C17—C18	119.82 (16)
C7—C6—C5	119.84 (16)	C16—C17—H17	120.1
C7—C6—H6	120.1	C18—C17—H17	120.1
C5—C6—H6	120.1	C13—C18—C17	119.50 (16)
C6—C7—C8	121.71 (16)	C13—C18—H18	120.2
C6—C7—H7	119.1	C17—C18—H18	120.2
C8—C7—H7	119.1	C12—C19—H19A	109.5
C7—C8—C9	120.46 (15)	C12—C19—H19B	109.5
C7—C8—C3	118.54 (16)	H19A—C19—H19B	109.5
C9—C8—C3	120.99 (15)	C12—C19—H19C	109.5
C10—C9—C8	121.70 (15)	H19A—C19—H19C	109.5
C10—C9—H9	119.2	H19B—C19—H19C	109.5
C8—C9—H9	119.2		
			
O2—S—C1—C12	136.48 (14)	C12—O1—C11—C2	1.42 (17)
O3—S—C1—C12	6.22 (17)	C12—O1—C11—C10	−177.49 (15)
C13—S—C1—C12	−108.55 (14)	C3—C2—C11—O1	179.31 (12)
O2—S—C1—C2	−48.68 (16)	C1—C2—C11—O1	−1.14 (17)
O3—S—C1—C2	−178.93 (14)	C3—C2—C11—C10	−1.8 (2)
C13—S—C1—C2	66.30 (16)	C1—C2—C11—C10	177.73 (15)
C12—C1—C2—C11	0.43 (17)	C9—C10—C11—O1	178.28 (15)
S—C1—C2—C11	−175.02 (12)	C9—C10—C11—C2	−0.5 (3)
C12—C1—C2—C3	179.84 (17)	C11—O1—C12—C1	−1.10 (18)
S—C1—C2—C3	4.4 (3)	C11—O1—C12—C19	178.68 (14)
C11—C2—C3—C4	−176.13 (15)	C2—C1—C12—O1	0.41 (18)
C1—C2—C3—C4	4.5 (3)	S—C1—C12—O1	176.24 (11)
C11—C2—C3—C8	3.3 (2)	C2—C1—C12—C19	−179.31 (19)
C1—C2—C3—C8	−176.09 (17)	S—C1—C12—C19	−3.5 (3)
C2—C3—C4—C5	−179.13 (15)	O2—S—C13—C18	−12.25 (14)
C8—C3—C4—C5	1.5 (2)	O3—S—C13—C18	115.62 (13)
C3—C4—C5—C6	0.5 (3)	C1—S—C13—C18	−129.41 (12)
C4—C5—C6—C7	−1.6 (3)	O2—S—C13—C14	172.06 (12)
C5—C6—C7—C8	0.7 (3)	O3—S—C13—C14	−60.06 (14)
C6—C7—C8—C9	−177.78 (17)	C1—S—C13—C14	54.91 (14)
C6—C7—C8—C3	1.3 (3)	C18—C13—C14—C15	0.9 (2)
C4—C3—C8—C7	−2.3 (2)	S—C13—C14—C15	176.46 (12)
C2—C3—C8—C7	178.25 (14)	C13—C14—C15—C16	−2.1 (2)
C4—C3—C8—C9	176.74 (15)	C14—C15—C16—C17	1.3 (3)
C2—C3—C8—C9	−2.7 (2)	C15—C16—C17—C18	0.8 (3)
C7—C8—C9—C10	179.50 (16)	C14—C13—C18—C17	1.1 (2)
C3—C8—C9—C10	0.5 (3)	S—C13—C18—C17	−174.55 (13)
C8—C9—C10—C11	1.1 (2)	C16—C17—C18—C13	−1.9 (3)

Symmetry codes: (i) −*x*+1, −*y*, −*z*.

### Hydrogen-bond geometry (Å, °)

**Table:**

*D*—H···*A*	*D*—H	H···*A*	*D*···*A*	*D*—H···*A*
C4—H4···O2	0.95	2.35	3.190 (2)	147
C18—H18···O2	0.95	2.46	2.869 (2)	106
C19—H19B···O3	0.98	2.55	2.926 (2)	103
C19—H19C···Cg3^i^	0.98	3.03	3.735 (3)	130
C16—H16···Cg3^ii^	0.95	2.88	3.761 (3)	155

Symmetry codes:  (i) −*x*+1, −*y*, −*z*; (ii) −*x*+2, −*y*+1, −*z*.
